# Mortality and quality of life in the five years after severe sepsis

**DOI:** 10.1186/cc12616

**Published:** 2013-04-16

**Authors:** Brian H Cuthbertson, Andrew Elders, Sally Hall, Jane Taylor, Graeme MacLennan, Fiona Mackirdy, Simon J Mackenzie

**Affiliations:** 1 Department of Critical Care Medicine, Sunnybrook Health Sciences Centre, Bayview Avenue 2075, Toronto, ON M4N 3M5, Canada; 2 Health Services Research Unit, University of Aberdeen, Health Sciences Building, Ashgrove Road, Foresterhill, Aberdeen, AB25 2ZD, Scotland, UK; 3 Intensive Care Unit, Aberdeen Royal Infirmary, Westburn Road, Foresterhill, Aberdeen, AB25 2ZN, Scotland, UK; 4 Scottish Intensive Care Society Audit Group, Information Services Division, NHS National Services Scotland, South Gyle Crescent 1, Edinburgh, EH12 9EB, Scotland, UK; 5 Intensive Care Unit, Royal Infirmary of Edinburgh, Edinburgh, EH16 4SA, Scotland, UK

## Abstract

**Introduction:**

Severe sepsis is associated with high levels of morbidity and mortality, placing a high burden on healthcare resources. We aimed to study outcomes in the five years after severe sepsis.

**Methods:**

This was a cohort study using data from a prospective audit in 26 adult ICUs in Scotland. Mortality was measured using clinical databases and quality of life using Short Form 36 (SF-36) at 3.5 and 5 years after severe sepsis.

**Results:**

A total of 439 patients were recruited with a 58% mortality at 3.5 years and 61% mortality at 5 years. A total of 85 and 67 patients responded at 3.5 and 5 years follow-up, respectively. SF-36 physical component score (PCS) was low compared to population controls at 3.5 years (mean 41.8 (SD 11.8)) and at 5 years (mean 44.8 (SD 12.7)). SF-36 mental component score (MCS) was slightly lower than population controls at 3.5 years (mean 47.7 (SD 14.6)) and at 5 years after severe sepsis (mean 48.8 (SD 12.6)). The majority of patients were satisfied with their current quality of life (QOL) (80%) and all patients would be willing to be treated in an ICU again if they become critically ill despite many having unpleasant memories (19%) and recall (29%) of ICU events.

**Conclusions:**

Patients with severe sepsis have a high ongoing mortality after severe sepsis. They also have a significantly lower physical QOL compared to population norms but mental QOL scores were only slightly below population norms up to five years after severe sepsis. All survivors would be willing to be treated in an ICU again if critically ill. Mortality and QOL outcomes were broadly similar to other critically ill cohorts throughout the five years of follow-up.

## Introduction

Severe sepsis is an increasingly common condition that is associated with high levels of morbidity and mortality as well as placing a large burden upon healthcare resources throughout the world [1-4]. The morbidity and mortality from sepsis remain high [2-4]. For those who survive severe sepsis, many will develop complications associated with significant long-term sequelae [5-11]. Perhaps unsurprisingly, health related quality of life (QOL) and activities of daily living (ADL) are frequently significantly diminished [5-12].

QOL is a very important outcome for patients after critical care and has been shown to be poor in all subgroups of critical illness compared to the general population at various time points [13-16]. Poor QOL leads to poor patient satisfaction and high health care resource utilization [17,18]. There has been little work following up patients with severe sepsis for mortality beyond three years and no work looking at changes in QOL over a five-year period. Only one study looking at ADL has followed up such patients for functional outcomes to this time point [9].

The aim of this study was to determine the mortality and QOL in patients in the five years after severe sepsis. As secondary outcomes of interest, we also wanted to study the patients' willingness to be treated in an ICU again if they become critically ill, the nature of their memories of the ICU, their satisfaction with their current QOL and the impact of the index illness on their place of residence and employment status.

## Materials and methods

This was a prospective cohort study of patients with severe sepsis with ethics approval granted by the Scottish multi-center research ethics committee and informed consent obtained from all patients' quality of life follow-up after three years. The ethics committee approval required the research team to make contact with patients indirectly through their parent hospital team, and first and only if the patients agreed, could the research team contact the patient. Mortality follow-up used national registry databases which are known to have an accuracy of 99%.

### Cohort and data collection

The study was of a prospective cohort of patients with severe sepsis collected between April and October 2003. Patients were identified as having: a.) evidence of three of four systemic inflammatory response syndrome (SIRS) criteria within the previous 24 hours, b.) confirmed or clinically strongly suspected infection, c.) two or more sepsis induced organ failures of less than 24 hours duration [19], and d.) an Acute Physiology and Chronic Health Evaluation (APACHE II) score greater than or equal to 25 based within 24 hours. Inclusion criteria were the criteria for activated protein C administration at the time of the study [20]. Data were collected in all 26 general adult ICUs in Scotland during the study period. Hospital mortality, ICU mortality, ICU length of stay and hospital length of stay were obtained from clinical ICU databases.

### QOL measures and outcomes

Patients completed Short Form-36 (SF-36) questionnaires and euroQOL-5D (EQ-5D) by telephone survey at 3.5 and 5 years after ICU admission and the results were calculated using standard techniques [13-16,21,22]. No data points were omitted from these surveys. QOL data were presented as standard deviations as appropriate. SF-36 mental component scores (MCS) and physical component scores (PCS) were calculated using standard methods and are presented on a normalized scale with 50 being the population norm and one standard deviation representing 10 points. The co-primary outcomes were mortality and QOL at five years. Secondary outcome measures included QOL at 3.5 years after ICU discharge; ICU, hospital and 3.5 year mortality; ICU length of stay and hospital length of stay. Questions related to other aspects of their ICU experience, satisfaction with QOL and changes in employment status were also asked.

### Statistical analysis

Data are presented as numbers and percentages, means and standard deviations or median and interquartile ranges as appropriate. For baseline sepsis and ICU data, numbers and percentages or median and interquartile ranges were calculated as appropriate. Differences in quality of life between 3.5 years and 5 years were compared using paired-tests.

### Role of the funding source

The study sponsor and funding sources had no role in the collection, analysis and interpretation of data; in the writing of the report; and in the decision to submit the paper for publication.

## Results

**Figure 1:**
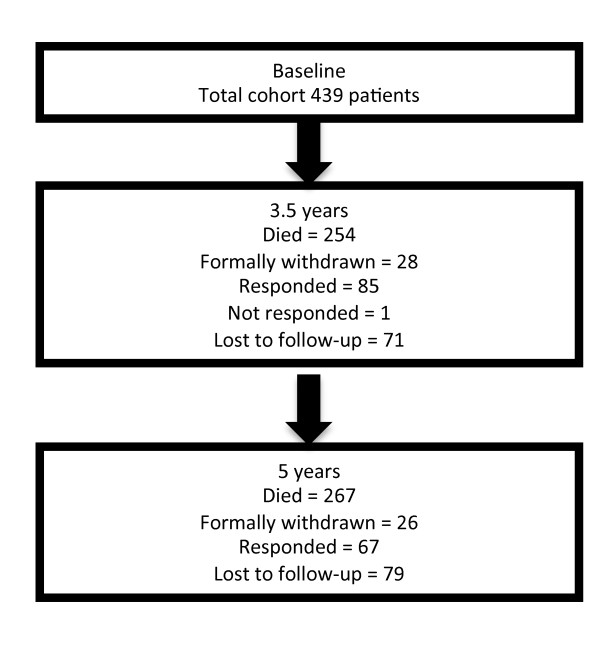
**Participant flow diagram**.

The flow of patients through the study is presented in Figure 1. Mortality follow-up was complete. The baseline, ICU and hospital characteristics for all groups are presented in Table 1. In summary of these data, the cohort had a median age of 58 (45 to 67), a high severity of illness (APACHE II = 23 (17 to 28); Simplified Acute Physiology Score (SAPS II) = 41 (30 to 54)) and a low level of chronic comorbidities (median 0 (0 to 0). The subjects were 53% male and had a range of organ system dysfunctions with respiratory failure being most common (65%). Infection was microbiologically confirmed in 35% of cases, with bronchopulmonary infection the most common source (48%), followed by other infections (21%) and blood infections (14%). Gram positive infection made up 16% and Gram negative infection (10%) of proven infections. Further data related to the severity, site and nature of sepsis are presented in Table 2.

**Table 1:** Baseline, intensive care and hospital characteristics of study patients.

	All cases	N = 439	
	**Median**	**25^th^**	**75^th^**

Age	58.0	45.0	67.0
SAPS II score	41.0	30.0	54.0
SAPS II predicted mortality	26.6	10.6	55.3
APACHE II score	23.0	17.0	28.0
APACHE II predictive mortality	46.0	23.6	66.5
Number of APACHE II comorbidities (median, IQR)	0	0	0
Number of APACHE II comorbidities (mean, SD)	0.2	0.5	
Length of hospital stay prior to ICU	1	0	3
ICU length of stay (all comers)	7.1	2.7	15.8

ICU length of stay (ICU survivors, n = 277)	8.8	3.9	17.9
ICU length of stay (ICU non-survivors, n = 162)	3.9	1.3	118
Hospital length of stay	19.0	8.0	41.2
	**n**	**%**	

Sex (male)	234	53%	
Readmission to ICU	9	2%	
Cardiovascular dysfunction	237	54%	
Respiratory dysfunction	282	65%	
Renal dysfunction	166	38%	
Metabolic acidosis	195	45%	
Haematological dysfunction	100	23%	

APACHE II system failure			
Respiratory	138	31%	
Cardiovascular	124	28%	
Neurological	44	10%	
Gastrointestinal	93	21%	
Other	26	7%	
Unknown	14	3%	
Activated protein C administration	94	21%	

Data presented as medians and 25^th ^and 75^th ^centiles or as number and percentages as appropriate.

**Table 2:** Data on severity, site and nature of sepsis for study patients

	All patients	N = 439
	**n**	**%**

APACHE II diagnosis of sepsis	72	16%
Microbiologically confirmed infection	155	35%
Site of infection		
Blood/bacteraemia	63	14%
Bronchopulmonary	211	48%
Deep wound	43	10%
Urinary	16	4%
Other	91	21%
Unknown	15	3%
Nature of infection		
Bacterial - Gram positive	69	16%
Bacterial - Gram negative	42	10%
Mixed	35	8%
Fungal	6	1%
Viral	7	2%
Unknown	280	64%

Data presented as numbers and percentages.

**Figure 2:**
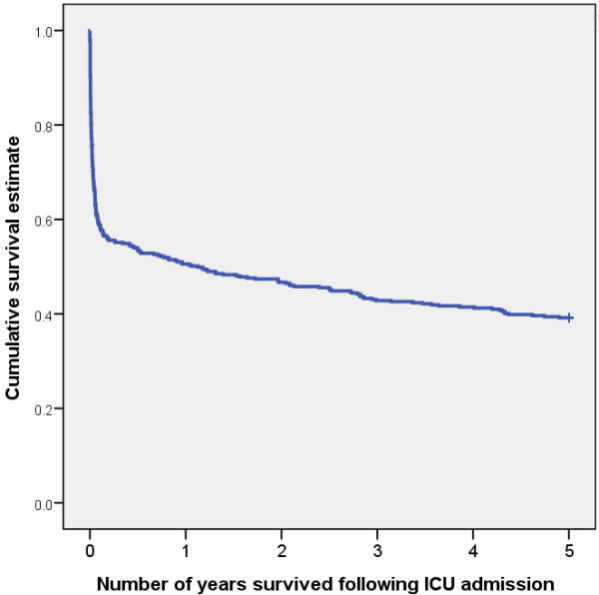
**Kaplan-Meier survival estimates for the entire cohort**.

The survival data show that 267 patients (63%) survived to leave the ICU, 249 (57%) survived to leave the hospital, 185 (42%) survived to 3.5 years and 172 (39%) survived to 5 years (Figure 2). Loss to follow-up occurred in 79/494 (16%) patients. Of the 185 survivors at 3.5 years, 28 withdrew and 72 were lost to follow-up, leaving 85 responders. Of the 172 survivors at five years, 26 withdrew and 78 were lost to follow-up, leaving 67 responders.

Table 3 shows the QOL outcomes including EQ-5D and SF-36 domains and component scores. EQ-5D scores are 0.64 (SD 0.36) at 3.5 years and 0.68 (SD 0.32) at 5 years. SF-36 PCS is lower than population controls at 41.8 (SD 11.8) at 3.5 years and 44.8 (SD 12.7) at 5 years. SF-36 MCS is 47.7 (SD 14.6) at 3.5 years and 48.8 (SD 12.6) at 5 years.

**Table 3:** Quality of life outcomes for severe sepsis at 3.5 and 5 years

QOL metric	3.5 years			5 years			Comparison between 3.5 and 5 years			
	
	N	Mean	SD	N	Mean	SD	N	Mean	**95% C.I**.	*P*-value
EQ-5D	83	0.64	0.36	66	0.68	0.32	64	0.02	(-0.04, 0.07)	0.546
SF-36 PCS	82	41.8	11.8	62	44.8	12.7	61	0.3	(-2.4, 3)	0.818
SF-36 MCS	82	47.7	14.6	62	48.8	12.6	61	2.4	(0.5, 4.2)	0.014
Physical Functioning	85	37.5	14.5	67	40.9	15.1	67	2.9	(0.9, 4.9)	0.005
Role Physical	84	41.7	13.5	66	44.6	13.7	65	2.5	(-0.1, 5.1)	0.057
Bodily Pain	84	48.0	12.8	65	49.9	11.9	64	1.1	(-1, 3.2)	0.284
General Health	84	43.6	13.3	65	45.7	13.3	64	1.2	(-0.7, 3.1)	0.206
Vitality	85	46.2	12.2	67	45.8	12.4	67	-1.0	(-3.3, 1.3)	0.380
Social Functioning	84	44.9	13.6	67	44.8	14.2	66	-0.1	(-2.2, 2)	0.938
Role Emotional	83	43.4	16.2	66	46.8	14.6	65	2.7	(-0.9, 6.2)	0.135
Mental Health	85	47.0	13.8	67	47.6	13.2	67	0.3	(-1.8, 2.5)	0.764

CI, confidence interval; MCS, mental component score; N, number; PCS, physical component score; SD, standard deviation; SF-36, short form 36.

At five years, the SF-36 PCS of 44.8 is significantly lower than the mean population norm of 50 (*P *= 0.001). The equivalent comparison for the mental component at five years shows no significant difference (*P *= 0.453).

At five years all patients stated they would be willing to be treated in an ICU again if they become critically ill, 19% had unpleasant memories of ICU events, 80% were either very happy or mostly happy with their current QOL, with 75% either working less or not working at all compared to before their critical illness (see Table 4).

**Table 4:** Other study outcomes

	3.5 years		5 years	
**Would you be willing to be treated in an ICU again?**	Valid N = 74		Valid N = 57	
***Yes***	74	100%	57	100%
***No***	0	0%	0	0%
**Do you have unpleasant recall of ICU events?**	Valid N = 84		Valid N = 66	
***Yes***	26	31%	19	29%
***No***	58	69%	47	71%
**Do you have unpleasant memories of ICU events?**	Valid N = 84		Valid N = 67	
***Yes***	12	14%	13	19%
***No***	72	86%	54	81%
**How do you rate your satisfaction with your current QOL?**	Valid N = 84		Valid N = 67	
***Very happy***	16	19%	13	19%
***Mostly happy***	51	61%	41	61%
***Often unhappy***	13	15%	12	18%
***Very unhappy***	4	5%	1	1%
**What is your current place of residence?**	Valid N = 79		Valid N = 63	
***Private home without assistance***	62	79%	49	78%
***Private home with assistance ***	16	20%	12	19%
***Sheltered housing***	0	0%	2	3%
***Care home***	1	1%	0	0%
***Other***	0	0%	0	0%
**What is your current employment status?**	Valid N = 66		Valid N = 55	
***Full time employment / studies***	19	29%	14	25%
***Part time employment / studies***	4	6%	5	9%
***Occasional employment***	0	0%	0	0%
***Retired***	29	44%	24	44%
***Unemployed***	12	18%	11	20%
***Long term disability***	2	3%	1	2%
**Has your employment status changed since your illness?**	Valid N = 84		Valid N = 65	
***Yes***	19	23%	12	18%
***No***	65	77%	53	82%
**If you were employed before your ICU admission, what is the intensity of your current employment compared to before your illness?**	Valid N = 62		Valid N = 58	
***I work more***	14	13%	7	0%
***No difference***	14	13%	15	25%
***I work less***	25	67%	24	50%
***I am not working***	9	7%	12	25%

## Discussion

This cohort study was designed to study mortality and QOL in the five years after ICU admission for severe sepsis and demonstrates a high mortality in this cohort of patients at this time point. The majority of this mortality occurs in hospital and within the first year but there is an ongoing attrition over the entire five-year period. This attrition has been demonstrated in other general ICU cohorts, but in sepsis cohorts follow-up has not previously been over such a long time period [14,15,23]. This study also demonstrates poor physical QOL scores in sepsis patients compared to age- and sex-matched population controls at 3.5 and 5 years. The QOL scores in this cohort are broadly similar to QOL scores up to five years in other cohorts of critically ill cohorts [14,15,17,24]. As has been previously demonstrated in other critically ill cohorts, mental QOL is only slightly below the age- and sex-matched population control level [12,14,15,17,24]. All our patients stated they would be willing to be treated in an ICU again if they become critically ill.

In a landmark study, Quartin *et al*. followed up survivors of severe sepsis for eight years and showed an excess mortality compared to population controls [11]. Other studies have looked at survival in severe sepsis subgroups, such as community-acquired pneumonia, but find the same pattern in excess long-term mortality [25-27]. A *post-hoc *follow-up of patients from the PROWESS (Recombinant Human Activated Protein C Worldwide Evaluation in Severe Sepsis) study showed that the reduction in mortality seen at 28 days was lost after hospital discharge through to 3.6 years although most patients in this study were followed up for shorter durations [28]. When we compare five-year mortality data for this group, we can look at results from general ICU [14,15,24,29] or acute respiratory distress syndrome (ARDS) patients [17]. All these studies demonstrate an ongoing attrition in these cohorts with time compared to age- and sex-matched population cohorts. These results seem broadly similar to severe sepsis mortality outcomes although the younger age on ICU admission in our cohort may explain the slightly lower than expected mortality rates between 3.5 and 5 years [9,11,25-27].

There have been a few papers looking at differences in QOL outcomes in severe sepsis patients. A study by Heyland *et al*. suggested that SF-36 QOL scores were lower than the general population and that SF-36 had a good reliability and validity in this group [10]. A study by Hofhuis *et al*. showed poor QOL scores at six months after an ICU stay compared to age- and sex-matched controls [5]. A study by Granja *et al*. also found poor QOL in survivors of sepsis that was comparable to other non-septic critical illness survivors [30]. A study by Longo *et al*. studied patients receiving activated protein C compared to unmatched controls and looked at mortality and QOL outcome up to seven months after ICU discharge [31]. They found an 11.8% absolute risk reduction for mortality in the activated protein C patients at seven months and improvements in a variety of QOL scores. This study was limited by small sample size and the lack of matching. Further studies have looked at ADL in severe sepsis survivors [9]. This study demonstrated that many severe sepsis patients have poor levels of activity (measured by ADL) in the period before severe sepsis and go on to have further functional disability in the five years after sepsis but not all of these patients are admitted to an ICU during their illness. The exact relationship between ADL and physical QOL in severe sepsis survivors is not fully understood but the information we elucidate from these metrics are likely to overlap to some degree.

The current study focuses on QOL outcomes up to five years after severe sepsis. There are no studies we have identified that studied QOL in severe sepsis patients for this long a time period. The most comparable studies are ones of general ICU cohorts [14,15,24] and of ARDS patients [17]. Broadly, these QOL scores are similar to those of other critically ill cohorts over this long follow-up [30]. It is interesting to note that the mental QOL scores are only slightly below the age- and sex-matched population control levels, which seems at odds with papers suggesting high levels of psychological morbidity and cognitive dysfunction after critical illness [9,12,15,17,24,32-35]. This may suggest a problem with the sensitivity or calibration of the SF-36 MCS in patients after critical illness since reports of depression, anxiety and post-traumatic stress disorders after severe sepsis and other critical illness seem so consistent [15,34-38]. Clearly, cognitive dysfunction is not measured in the SF-36 MCS *per se *but it may confound the measurement of both mental and physical QOL scores.

It was very interesting to find that all our patients would be willing to undergo treatment in an ICU again if they become critically ill despite generally poor physical QOL scores and 19 to 29% having either unpleasant recall or memories of their ICU stay [37,39,40]. Despite the relatively poor QOL scores, the vast majority (80%) of patients reported they were mostly or very happy with their current QOL [41]. Of course, there could be a cognitive bias in these responses as it must be difficult to state you would not want life saving treatments or that you have a poor QOL. These data also seem to somewhat contradict the results of previous work in acutely ill patients [40]. With regard to employment status, 34% were in full time employment at five years with 75% stating they now work less or are now not working, since their illness [17,18,32]. Although it must be identified that some of this reduction in employment is due to retirements. This change in employment status speaks to the broad long-term financial impact of severe sepsis on patients and their families [15,18,42].

This study has a number of strengths and limitations that need to be considered. Strengths include the multi-center nature of the data and the fact that all adult general ICUs in one country were included. Further strengths include the prolonged follow-up period that has not been attained in previous studies. Compared to previous studies in this field, this is a reasonable sized study, but from an epidemiological perspective the limited patient number reduces the ability to make strong conclusions about this cohort. Limitations include the loss to follow-up in this study. The cohorts were not approached for consent for long term follow-up until well after their index ICU admission, thus denying us shorter term QOL data. The ethics committee approval required the research team to make contact with patients indirectly through their parent hospital team first and only if the patients agreed could the research team contact the patient. We believe that this additional step led to a significantly lower recruitment and retention to the study than a direct approach would have achieved. This has the potential to introduce a recruitment bias into the study data collection, although we are unable to identify the magnitude of direction of such a bias. Since it is likely that poor QOL is more common in those patients who die during follow-up, there is likely excess QOL loss to follow-up in the highest risk patients and, therefore, these QOL results may, on average, be better than those actually experienced by the patients. This issue is standard to all studies using prospective cohort designs. With regard to the generalizability of the results, we recruited patients from all adult ICU patients in one country which enhances the generalizability but we only recruited patients with an APACHE II score ≥25 making this a sicker cohort than in some studies of severe sepsis. It is noted that despite the inclusion criteria for this cohort being APACHE II ≥25, some patients with lower APACHE II scores were recruited to the study. In this study we demonstrated that 35% of infections were microbiologically confirmed compared to those in European Prevalence of Infection in Intensive Care (EPIC) and 60% in Sepsis Occurrence in Acutely Ill Patients (SOAP) studies [43,44]. A further limitation is the lack of pre-morbid data on physical or psychological morbidity and function [9,34,45]. Recent studies have identified the high level of pre-morbid physical, psychological and cognitive dysfunction in this group and that levels of these morbidities may not always be significantly higher than in the post-morbid recovery phase of critical illness [9,34,45]. We are, therefore, unable to confidently determine the acuity of these symptoms or their relationship to the current levels of dysfunction to the index illness in our patient group. Finally, it has been suggested by one author that QOL data should be adjusted for pre-morbid conditions, we did not do this as we believe that this is not an accepted methodological standard for QOL measurement [46].

## Conclusions

Patients with severe sepsis have a high ongoing mortality after severe sepsis with only 61% surviving five years. They also have a significantly lower physical QOL compared to the population norm but mental QOL scores were only slightly below population norms up to five years after severe sepsis. Mortality and QOL outcomes were broadly similar to other critically ill cohorts throughout the five years of follow-up. These data need to be considered when evaluating longer-term outcomes and when considering the cost-effectiveness of care in this patient group.

## Key messages

• Sepsis is known to have a high, shorter-term mortality; this high mortality seems to continue for up to five years after severe sepsis.

• Quality of life is known to be poor in the years after critical care admission and we have demonstrated similar patterns of QOL deficit after severe sepsis.

• The majority of severe sepsis survivors were satisfied with their current QOL and all patients would be willing to be treated in an ICU again if they become critically ill despite many having unpleasant memories and recall of ICU events.

## Abbreviations

ADL, activities of daily living; APACHE II, Acute Physiology, Age And Chronic Health Evaluation; ARDS, Acute respiratory distress syndrome; EPIC, European Prevalence of Infection in Intensive Care; EQ-5D, euroQOL-5D; ICU, intensive care unit; MCS, mental component score; PCS, physical component score; QOL, quality of life; SAPS, Simplified Acute Physiology Score; SD, standard deviation; SF-36, short form 36; SIRS, systemic inflammatory response syndrome; SOAP, Sepsis Occurrence in Acutely Ill Patients

## Competing interests

This study was funded by the Scottish Intensive Care Society and an unrestricted research grant from Eli Lilly and Company. Eli Lilly and Company did not have input into the study design or analysis and did not approve the final results. Dr Brian Cuthbertson has received honoraria for presentations and for consultancies from Eli Lilly and Company. There are no other financial or non-financial competing interests for this manuscript.

## Authors' contributions

BHC, FM and SJM have participated fully in the design of this study, the collection and analysis of data and in the writing of the paper, and have seen and approved the final version of the paper. AE and GM have participated fully in analysis of the data and in the writing of the paper, and have seen and approved the final version of the paper. SH and JT have participated fully in the collection of data, analysis of the data and in the writing of the paper, and have seen and approved the final version of the paper.
